# Dermatology and COVID-19: The Hidden Pandemic

**DOI:** 10.3390/jcm11154397

**Published:** 2022-07-28

**Authors:** S. Recalcati, G. Nazzaro

**Affiliations:** 1 Dermatology Unit, ASST Lecco, Alessandro Manzoni Hospital, Via dell’Eremo 9/11, 23900 Lecco, Italy; 2 Dermatology Unit, IRCCS Ca’ Granda Foundation Ospedale Maggiore Policlinico, 20122 Milan, Italy; gianluca.nazzaro@gmail.com

Since December 2019, a novel coronavirus, known as severe acute respiratory syndrome coronavirus 2 (SARS-CoV-2), has been rapidly spreading across the world, leading to the declared pandemic of COVID-19. A strong effort to fight against this new disease was required from every physician, and a virtuous collaboration involving all specialties was established [1]. Physicians routinely predestined to different tasks were redeployed to the frontlines into the COVID departments [2]. A few months into the crisis, dermatologists began to describe cutaneous manifestations likely related to this virus [3], and scientists worldwide became aware of this correlation. Indeed, the recognition of cutaneous manifestations in asymptomatic patients could be helpful for epidemiological control, especially in areas where diagnostic tests are scarce [4].

As many asymptomatic patients did not seek medical consultation for skin eruptions, it was difficult to establish the real prevalence of COVID-19-related cutaneous manifestations. Considering the current literature, the prevalence varies from 5% to 18% [5]. Although the spectrum of skin manifestations is heterogeneous, physicians have tried to group them in different settings. We have also proposed our own classification for the use of both medical and paramedical personnel. Three main groups were described: exanthems, usually simultaneous with the onset of the extracutaneous COVID-19 symptoms; a second subset consisting of vascular lesions arising later and lasting longer; and a third miscellaneous group of different cutaneous manifestations, not directly linked to the viral action on the skin [5]. Over time, more histological, immunohistochemical, and laboratory data were collected, allowing the study of the pathogenetic mechanisms underlying the disease. For example, the vascular lesions are probably due to an unbalanced coagulation state and might represent a COVID-19-specific complication. We now know that SARS-CoV-2 can induce a prothrombotic state through different mechanisms, i.e., inflammation, platelet activation, and endothelial dysfunction, and this explains the possible formation of microthrombi in the dermal vessels [6]. Other important studies were conducted on chilblain-like lesions typically affecting children and young adults and usually yielding negative serology. The role of type I interferon was investigated, and its increase may indeed explain the mild disease in this particular population subgroup and the negativity of serology [7]. Moreover, some authors proposed that a vigorous innate immune response against the virus in this subset can control the infection without effectively generating antibodies through the adaptive response [8]. Studies on chilblain-like lesions continue, and we are still far to attribute them with certainty to COVID-19 [9].

Another aspect of dealing with dermatology was the emergence of numerous cutaneous adverse effects due to personal protective measures, i.e., gloves, masks, and sanitizers [10]. Most of them were new dermatologic manifestations, but in several cases, aggravation of pre-existing skin conditions was documented [11]. In particular, facial dermatoses [12], such as acne, rosacea, and seborrheic dermatitis [13], were reported and a newly coined term, deriving from the contraction of mask-related acne, was proposed to describe a form of mechanical acne resulting from continuous textile-skin adherence and friction [14]. Studies focusing on minimizing adverse effects will be important in maintaining compliance with these preventive measures alike to help with global efforts against the COVID-19 spread.

With the advent of vaccines, a new chapter of skin manifestations arising after their administration was described [15]. It is of interest that many manifestations were quite similar to those caused by the virus itself. Conversely, others were new, e.g., the delayed localized reactions dubbed the “COVID arm” [16]. Their timely recognition and treatment are essential to encourage patient compliance with the vaccination strategy.

The last consideration concerns the role that teledermatology has achieved during the time of this pandemic [17]. Its implementation has limited patients’ access to hospitals, which contributes to keeping the potential risk of contagion at low levels. Unfortunately, it had a negative impact on the early diagnosis and treatment of oncological diseases [18]: we have to be aware of its important limitations.

While the fight against the COVID-19 pandemic continues with the discovery of new treatment strategies [19], dermatologists worldwide are facing the hidden pandemic of COVID-19-related dermatological conditions.
